# Integrating insects in circular food systems: evidence, gaps and research priorities

**DOI:** 10.7717/peerj.21419

**Published:** 2026-07-10

**Authors:** Åsa Berggren

**Affiliations:** Department of Ecology, Swedish University of Agricultural Sciences, Uppsala, Sweden

**Keywords:** Ecology, Food, Ecosystem services, Nutrients, Frass, Recycling, Microbiota, Plants

## Abstract

**Background:**

Many of today’s food production systems follow a linear model, where natural resources are extracted, converted into food, and discarded as waste. Efforts to reduce the environmental impact of this model have primarily focused on improving nutrient conversion efficiency. However, because linear systems are open-ended, scaling up production typically results in increased environmental costs, making sustainability goals increasingly difficult to achieve. By contrast, circular food systems (CFS) aim to recycle internal waste streams, using intermediary organisms to transform organic waste into resources that can be reused within the system. Insects are particularly promising in this context due to their dual ecological role: they convert plant and other waste into edible biomass and produce frass—a residual waste product with fertilisation potential for supporting plant growth.

**Methodology:**

To assess the current state of knowledge, a structured literature review with a systematic search of peer-reviewed studies was conducted. The Web of Science Core Collection was used to search for two topic queries (“circular food system AND insect” and “circular food production system AND insect”) and included all peer-reviewed articles published through December 2024. This resulted in 395 articles identified, of which 63 met the inclusion criteria, focusing on insect integration within circular food systems.

**Results:**

Although insects are being actively studied in linear systems for their efficient biomass conversion and nutrient-rich outputs, their central role in circular food systems has so far been explored mostly through theoretical and modelling studies, with limited empirical validation. Unlocking their full potential in circular food systems requires a deeper understanding of how insects process waste, generate harvestable nutrients, and interact with other organisms in closed-loop conditions. Three urgent research priorities were identified: understanding the suitability of residual waste streams as insect feed, elucidating insect ecology and life-cycle dynamics under circular conditions, and exploring multi-species interactions within integrated systems.

**Conclusions:**

Because circular systems must function as simplified ecosystems with producers, decomposers, and consumers interacting across trophic levels, an ecosystem engineering approach will be needed to design and maintain them. This transition demands transdisciplinary collaboration and new ecological insights. Addressing these gaps is essential for realising the full ecological and functional potential of insects in sustainable circular food production.

## Introduction

With the global population projected to reach 10 billion by 2050 (FAO, 2017), there is growing concern that current agricultural practices will be unable to sustainably meet future food demands (Tilman et al., 2011; Van Dijk et al., 2021). Many current food production systems incur substantial environmental costs, including deforestation and land degradation, which threaten biodiversity, ecosystem services, and traditional livelihoods (Rockström et al., 2017). To support future generations, a transition toward sustainable food production is urgently needed and one that provides sufficient nutrition while preserving ecological integrity (Kennedy et al., 2020; Hansson et al., 2024). Many food systems today follow a linear ‘take - make –dispose’ model, where resources are extracted, used for production, and then discarded as waste (Poore & Nemecek, 2018; Kolawole et al., 2024). This linear food system results in resource inefficiency and exacerbates environmental degradation. In contrast, a circular food system (CFS) aims to minimise external inputs and emissions by recycling nutrients, water, and energy (Simon et al., 2024; Rahmann & Grimm, 2021; Roos et al., 2021). These integrated systems have the potential to reduce land use, enhance ecological sustainability, and close resource cycles within food production. Current sustainability challenges highlight the need to explore ecologically grounded strategies for resource cycling in food production. Conceptual work on circular ‘blue–green’ bioeconomies similarly explores how terrestrial and aquatic production systems can be jointly considered by valorising residual streams across sectors (Banovic et al., 2025), providing one example of how circular principles can be applied across domains. Current sustainability challenges therefore highlight the need to explore ecologically grounded strategies for resource cycling in food production.

Insects are of particular interest in this context due to their ecological functions and system-level potential. They can convert byproducts into edible biomass (food and feed), support nutrient cycling, and transfer nutrients across trophic levels. These roles are also played by microbes and fungi, insects offer added advantages in scalability and nutritional output. While other organisms (*e.g.*, microbes, fungi) contribute to these functions, insects are especially interesting due to their increasing adoption in alternative protein systems. Although insects have received growing attention for their role in feed and food production, their integration into empirically validated CFS remains underexplored. In this review, I critically assess both empirical and theoretical studies to identify current knowledge gaps and define ecological research priorities for their system-level integration. This review is directly relevant to researchers in circular food system design and ecological engineering, insects as food and feed and to associated fields such as agronomy, soil and microbial ecology, waste management and food-systems modelling. Here, “insects in circular food systems” denotes research on how edible and decomposer insects mediate waste conversion and nutrient flows within designed food systems that minimise external inputs.

### Ecological framing of insects in circular food systems

**Figure 1:**
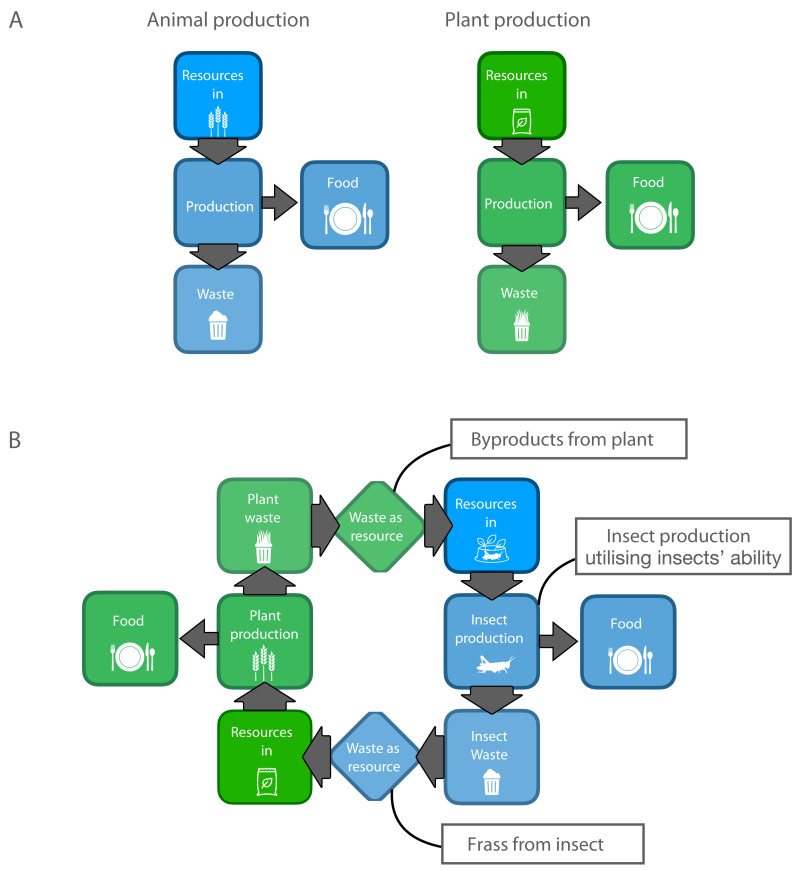
Conceptual comparison of linear and circular food production systems, showing how internal resource recycling can improve system efficiency and reduce waste. Schematic representation of food production systems. (A) Linear systems rely on large external resource inputs and generate substantial waste, resulting in low production efficiency relative to inputs. (B) Circular systems can interconnect edible insect production, plant cultivation, and other organisms through internal resource flows. Waste produced within the system is recycled and reused to support other components.

Insects have been proposed as key actors in circular food systems due to their dual function as waste recyclers and nutrient providers (Siddiqui et al., 2023). In the sections below, I outline their ecological contributions and explore the structure of CFS in which they may be integrated. To do so, I describe the basic structure of food systems in terms of production components (*i.e.,* organisms grown for nutrient harvesting) and their linked input and output flows. Linear systems rely heavily on external inputs (*e.g.*, energy, water, nutrients) and generate substantial waste relative to their food output (Fig. 1A). This imposes burdens on surrounding ecosystems for both resource extraction and waste assimilation. Although improved feed conversion efficiency (*e.g.*, through optimised breeds or species) can reduce some of these impacts (Chewning et al., 1990; Haroon et al., 2022), scaling up linear systems still leads to escalating ecological costs.

**Figure 2:**
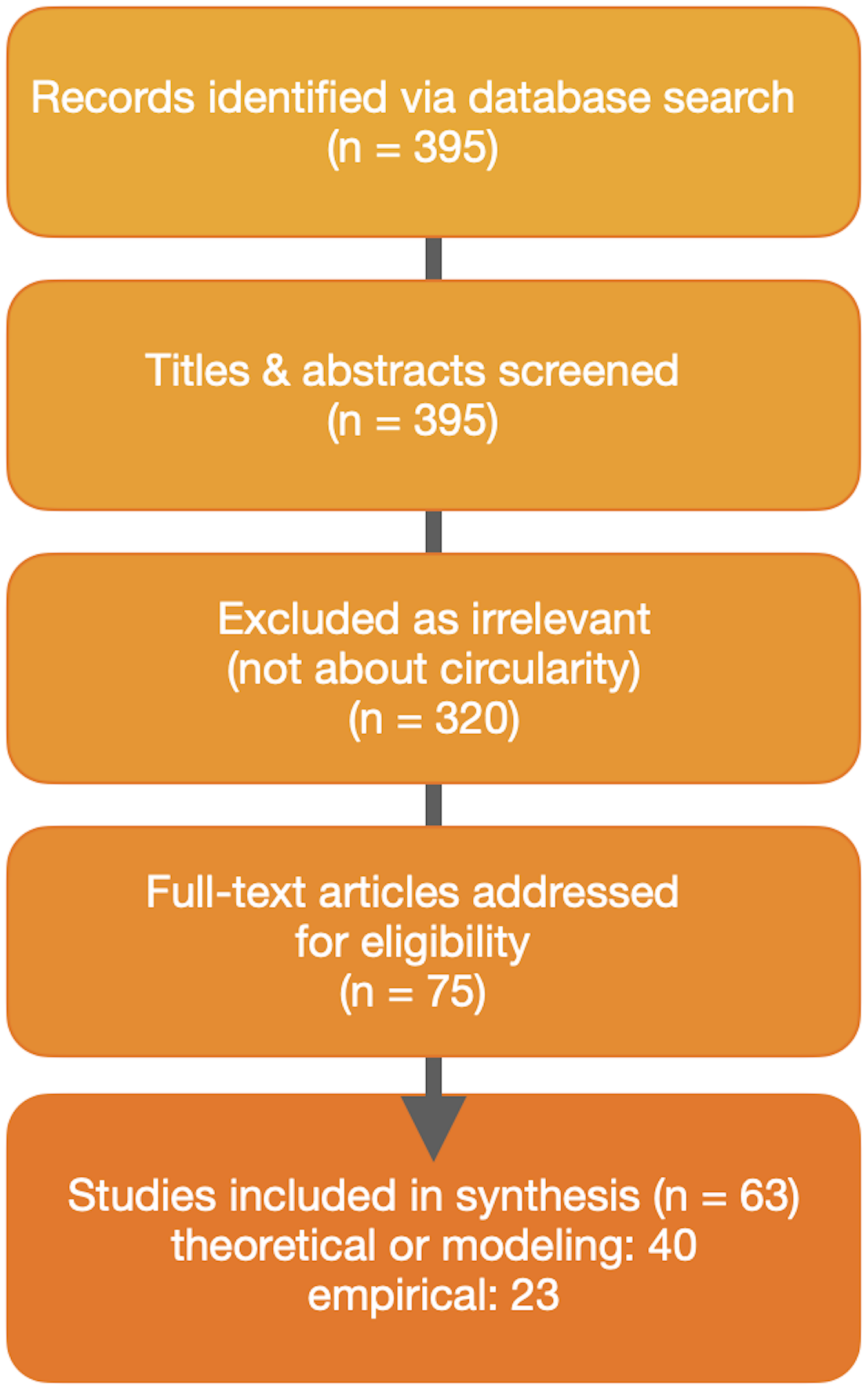
PRISMA-style flow diagram illustrating the identification, screening, and selection of studies included in this review of insects in circular food systems.

A more sustainable alternative is to develop circular systems that derive most of their resource inputs internally by recycling biological waste (Fig. 2B; Toplicean & Datcu, 2024). Despite longstanding discussion of circular production in economics (Ghisellini, Cialani & Ulgiati, 2016), the ecological science underpinning circular food systems is still in its infancy. In this review, the term “waste” is used to refer to organic residual streams from the food supply chain, from primary production to post-consumer disposal. In animal production, examples of waste include animal excreta (*e.g.*, manure), spent bedding and litter. In plant production, such wastes include crop residues (*e.g.*, stems, stalks), processing by-products (*e.g.*, pulps, peels), spoiled or surplus produce. It is important to note, however, that not all of these organic residual streams are necessarily discarded or treated as waste. For example, crop residues from field-grown crops are often incorporated back into the soil to build organic matter, although their benefits may vary with climate and soil conditions (Turmel et al., 2015). Similarly, animal manure is widely applied as fertiliser in crop production, although in regions with high livestock densities surpluses can exceed the assimilative capacity of surrounding farmland, leading to nutrient imbalances and environmental risks. While many streams are already reused within food systems, their broader potential, such as transformation into high-value inputs through novel pathways remains underdeveloped, and in many cases largely theoretical (Sandström & Kummu, 2023).

### The case for insects in circular systems

A key requirement of a CFS is the presence of organisms that can transform organic waste into usable resources for other components of the system. These include microorganisms, fungi, and insects—taxa that naturally perform such roles in ecosystems. Microbes and fungi decompose organic matter and contribute to soil fertility (Pii et al., 2015), while many insects efficiently convert plant-derived waste into both insect biomass and nutrient-rich frass (Van Huis et al., 2013). Members of these taxa are promising candidates for circular food production, but their integration requires ecological knowledge of how their outputs (*e.g.*, biomass or frass) can be harvested as food or used as feed within the system. Although the review focuses on studies explicitly framed within a CFS, it builds on a broader ecological foundation to evaluate how insects can support nutrient transformation and enhance resource efficiency in redesigned food systems. I focus on insects due to their proven scalability, established safety as food and feed, and their ability to convert waste into edible insect biomass and nutrient-rich frass.

Novel foods such as algae, fungi, and insects have gained attention for reducing inputs in linear food systems (Wells et al., 2017; Troiano, Orsat & Dumont, 2020; Siddiqui et al., 2023). Insects are recognised for their high feed conversion efficiency, meaning they require significantly fewer inputs to produce equivalent biomass compared to conventional livestock (Nakagaki & De Foliart, 1991; Wilkinson, 2011). The nutrient profiles of many edible insect species—rich in protein, fatty acids, and essential minerals—further support their role in sustainable diets (WHO , 2007; Finke & Oonincx, 2017) and have fuelled a recent global expansion of insect production (Jansson, Hunter & Berggren, 2019). However, most current insect applications target replacement of livestock within linear food systems (WHO , 2007; Van Huis et al., 2013). Their potential role in transforming food systems from linear to circular has received comparatively little attention (but see Berggren, Jansson & Low, 2019; Hamam, D’Amico & Di Vita, 2024). Insects can convert residual streams that are typically treated as waste (Ojha, Bußler & Schlüter, 2020; Koutsogeorgiou et al., 2024; Kuo & Fisher, 2022) into nutrient-rich biomass for human consumption (Miech et al., 2016; Miech et al., 2017; Torok, Luyckx & Lapidge, 2021; Magara, Hugel & Fisher, 2024), feed for livestock, and frass for crop production. Several species such as the house cricket (*Acheta domesticus*) and yellow mealworm (*Tenebrio molitor*) have EU safety approval for human consumption (EU, 2022; EU, 2023). Many more insects are already part of established diets in different parts of the world (Gómez-Corona & Valentin, 2023), and some can have their nutrient composition manipulated through feed substrates (Van Huis et al., 2021). These edible species can be integrated into CFS, both as waste bioconverters and as sources of nutrients for humans and other organisms (Fig. 1B).

### Ecological interactions and functions in circular systems

Understanding ecological interactions is essential to designing stable and functional CFS. It is the stability and complementarity of interactions among organisms that governs how resources circulate, waste decomposes, and nutrients flow within the system. For example, plant–microbe interactions can enhance nutrient uptake and protect plants against pests (Mendes, Garbeva & Raaijmakers, 2013; Mauchline & Malone, 2017), while insect frass can stimulate microbial activity and contribute to soil health (Houben et al., 2020). These interspecies interactions are fundamental to the ecological functioning of CFS but remain poorly understood. Despite their clear relevance, insect-driven ecological interactions within circular food systems have not been systematically examined. As a first step toward incorporating insects as functional ecological agents in CFS, I synthesise existing knowledge from studies where insects are explicitly integrated into circular frameworks.

## Survey Methodology

### Literature search

I conducted a structured literature review to identify studies examining the role of insects in CFS, with particular focus on their ecological functions, system integration, and capacity to recycle waste. The primary search was conducted in the Web of Science Core Collection, using two topic queries: “circular food system AND insect” and “circular food production system AND insect”. The search included all peer-reviewed articles published through to December 2024.

### Screening and inclusion criteria

The initial search retrieved 395 unique records. Titles and abstracts were screened using the following inclusion criteria: (1) the study explicitly addressed food systems incorporating circular design principles, and (2) insects were integrated as nutrient converters, system components or ecological agents. Studies that focused solely on insects as a feed component without a broader system-level integration were excluded. This screening yielded 75 articles for full-text review. The screening and selection process is illustrated in Fig. 2.

### Eligibility assessment and study classification

During the full-text eligibility assessment, articles were evaluated for their contribution to at least one of the following areas: (a) insect-based waste conversion, (b) within-system nutrient cycling, or (c) ecological interactions involving insects within CFS. Based on these refined criteria, 63 studies were retained. A PRISMA-style checklist is provided as Table S1 to support methodological transparency. No formal statistical analysis was applied; the review was based on structured screening, qualitative content assessment and descriptive classification of the included studies by research focus, study type and ecological function.

### Scope and delimitation

This review focused specifically on peer-reviewed, English-language publications indexed in the Web of Science Core Collection that explicitly framed their research within the CFS paradigm. While this approach ensured consistency and alignment with CFS principles, it may have excluded relevant studies from broader ecological fields (*e.g.*, insect–microbe–plant interactions) that contribute indirectly to circularity but do not self-identify with CFS terminology. Grey literature and preprints were also not included in the search, potentially limiting coverage of the most recent or practice-based work. The scope of the review remains intentionally bounded by the inclusion parameters to assess targeted progress within a CFS framework. Within these boundaries, the use of a structured Web of Science search, predefined inclusion criteria and PRISMA-style reporting was intended to maximise coverage of relevant studies and minimise subjective selection bias.

## Results

### The state of knowledge for incorporating insects into circular food systems

Of the total 63 studies in the analysis (Article S1), only 23 were empirical (Table S2) and 40 were theoretical or modelling-based contributions. Of the empirical studies, 13 examined the use of different byproducts (*e.g.*, fruit and vegetable waste) as feed for insects (Chineme et al., 2023; Ribeiro, Costa & Ameixa, 2022). Not all experiments examined biological responses in insects; only 10 studies examined, for example, survival rate or growth of individuals. Five of the studies examined the use of insect frass as fertiliser, where one study examined the nutrient content of nine different species. None of the studies examined interactions between species (other than as feed) in the system. The most common focus species in the studies were the black soldier fly (*Hermetia illucens*) (16 studies) and yellow mealworm (*Tenebrio molitor*) (5 studies), all other species such as house cricket (*Acheta domesticus*) and the giant mealworm (*Zophobas atratus*) were only present in single studies. The potential of insects as key components in a CFS was considered in several theoretical studies (Derler et al., 2021; Jagtap et al., 2021; Rahmann & Grimm, 2021; Shafer et al., 2022), but empirical evaluations of their combined ecological functions within integrated systems were absent. Below, I summarise current knowledge across four key domains: (1) insect-based waste conversion (2) insect ecology and nutrients, (3) within-system resource flows, and (4) organismal interactions.

### Insect-mediated waste conversion

One of the few empirical studies focused on CFS is Beesigamukama et al. (2022). This study demonstrated that *H. illucens* can grow on organic waste (brewery spent grain) and its frass can effectively be used as a fertiliser. This was the only study identified within a circular system that integrated insect-based waste conversion and another trophic level.

### Insect growth and life-history dynamics in circular systems

Even though 10 of the studies examined some aspects of individual responses to feed (*e.g.*, Ebeneezar et al., 2021; Lalander & Vinnerås, 2022), none of the empirical studies examined the full life-cycle performance of the insects, including growth rates, reproductive viability, and nutrient composition under circular system conditions. These are biological outputs that are expected to depend heavily on the types and quality of waste used as feed (Vaga, Berggren & Jansson, 2020a; Vaga et al., 2020), but their suitability for sustaining insect populations in CFS remain unknown. Current knowledge is derived almost entirely from studies on insect feeding in linear systems using agricultural byproducts (Acur, Malinga & Nyeko, 2024; Vrontaki et al., 2024).

### Insect byproducts and nutrient recycling

Only five experimental studies were identified that assessed how frass could be reused as inputs for crop production within CFS (*e.g.*, Szopa et al., 2023; Wang et al., 2023). Frass constitutes a major waste stream in insect production and holds promise as a soil amendment. Several studies have demonstrated that frass is nutrient-rich (Amorim et al., 2024) and can enhance plant growth (Ojha, Bußler & Schlüter, 2020; Cammack et al., 2021; Poveda, 2021; Barragán-Fonseca et al., 2022; Andrianorosoa Ony et al., 2024) and recent work also shows that pre-treatment and application methods can strongly influence crop responses to frass fertilisation (Capitán, Berggren & Weih, 2025). Others suggest that frass can reduce susceptibility to abiotic and biotic stressors, such as drought and plant pathogens (Quilliam et al., 2020; Poveda, 2021). These studies were not within a fully integrated CFS where insects are reared on residual streams and their frass is returned to crop production as part of a connected system.

### Interactions of organisms in the system

I found no empirical studies addressing multi-species interactions within a CFS involving insects. Nearly all studies focused on single-species scenarios or theoretical frameworks (*e.g.*, Moruzzo et al., 2021; Derler et al., 2021; Cattaneo, Meneguz & Dabbou, 2023). Yet, species interactions, especially among insects, plants, microbes, and decomposers are likely to play critical roles in nutrient flow, stability, and system resilience. Soil microbial communities, for example, may drive enhanced plant growth and herbivore resistance (Blakstad et al., 2023). Recent studies also show that microbial abundance and community diversity are influenced by frass, with variation depending on the insect species used and the frass composition (Xu et al., 2023; Van De Zande et al., 2024). While these findings offer important ecological clues, they have not yet been tested in CFS with interacting organisms. Together, these findings reveal a limited but emerging body of work on insect roles in circular food systems, with major gaps in empirical research on ecological integration.

## Discussion

### Suitability of waste streams as food sources

Despite frequent claims about their potential, only a limited number of empirical studies have examined insect feed conversion in explicitly circular contexts using residual waste streams. This mismatch between the theoretical promise of residual materials and the scarcity of system-level evidence defines a first research priority: systematically identifying which residual streams can reliably function as insect feed within CFS. Existing studies nonetheless indicate that residual materials such as food waste and agricultural byproducts hold significant promise as feed in a CFS (Siddiqui et al., 2023). However, these waste streams vary widely in composition, quality, and availability, making it essential to understand which materials are suitable for insect feed. Detailed knowledge about waste-specific nutrient profiles, along with how materials are harvested, handled, and pre-treated, is critical to determining their utility as insect feed. Although some residual streams, such as crop residues and manure, are already reused in food systems (Turmel et al., 2015; Sandström & Kummu, 2023), these pathways are only partially circular, underscoring the need to determine under which conditions insect-mediated bioconversion should complement or replace them within CFS.

**Figure 3:**
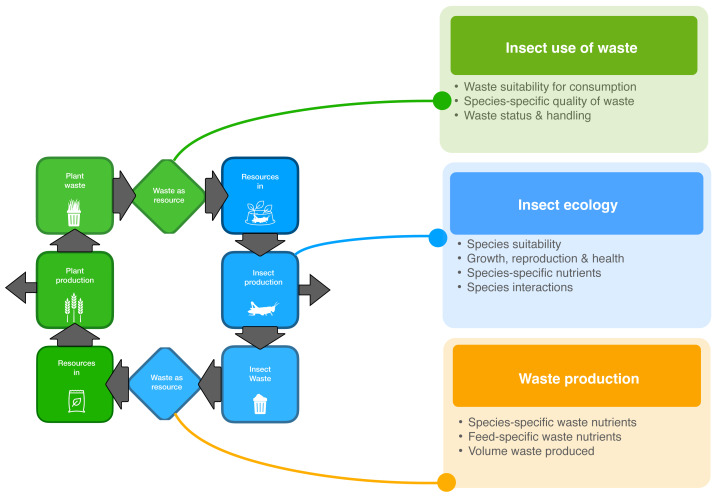
Key ecological knowledge gaps for integrating insects into circular food systems, including plant waste utilisation, species-specific ecological traits, and frass production as a within-system resource.

Building on this, one of the most urgent research priorities is to evaluate the ability and efficiency of different insect species to utilise diverse materials as feed sources. While prior studies have examined insect feeding on agricultural byproducts in linear systems (Miech et al., 2016; Montalbán et al., 2023; Gourgouta et al., 2024), future work must assess these processes within a CFS. In practical terms, this general priority can be translated into a set of specific, testable questions about how insects process real waste streams under circular conditions. Key research questions (see Fig. 3) emerging from this gap include:

• How efficiently can insects convert specific waste streams into harvestable food?
• What insect densities are needed to process waste volumes over time, and under varying environmental conditions (*e.g.*, temperature, humidity)?
• How does feed influence the nutritional quality of the resulting insect biomass for human (or animal) consumption?
• How are important biological parameters such as reproduction in insects affected by the factors above?
• What is the composition and fertilisation potential of frass produced from different waste?

Collectively, addressing these questions would directly test the assumption that residual streams can reliably be upgraded into edible biomass and fertiliser within CFS, rather than only in linear or partially circular systems. At the same time, frass constitutes a waste stream, that may be repurposed as a soil amendment for primary producers within a CFS (see also Fig. 2). Recent work highlights both its potential as a plant fertiliser and the importance of understanding its conditioning, application rates and possible risks in agricultural use (Hénault-Ethier et al., 2024). However, both waste inputs and the resulting insect-derived waste outputs can have incomplete or unbalanced nutrient profiles. This raises further questions about whether single-species systems (insects or plants) are sufficient, or whether multi-species approaches are needed to achieve balanced nutrient flows and optimise waste cycling (Derler et al., 2021). Additional factors such as seasonal availability, storage, processing, and transportation logistics of waste streams may also influence feed quality and affect bioconversion outcomes (Ho et al., 2024). Furthermore, the use of waste streams as inputs in a CFS should be evaluated not only for their productivity, but also for their broader environmental impacts. This includes assessing potential energy demands, carbon footprints and system-level trade-offs using life cycle assessment tools (Smetana, Spykman & Heinz, 2021; Nikkhah et al., 2024; Santolin et al., 2024). Ultimately, how insects respond to these variable feed inputs depends not only on their biology, but also on the rearing systems in which they are housed.

### Insect biology and rearing conditions in circular food systems

Insects must be reared efficiently and reliably under CFS conditions, which requires understanding their biological responses to feed, environment, and system dynamics. Here, I outline key questions at the levels of species selection, life cycle management, and rearing infrastructure. However, empirical studies on insect performance in circular systems are still scarce, highlighting a fundamental research need is to better understand how environmental conditions influence population performance. These challenges are not unique to CFS, they also apply to the emerging insects-as-food industry more broadly, which is still dominated by linear production models (Berggren, Jansson & Low, 2019). Several general questions require urgent attention for successful insect integration into food systems:

• Should we continue to rely on the most commonly reared species—such as house crickets (*A. domesticus*) and yellow mealworms (*T. molitor*)—or are there any alternative species with superior nutritional profiles, feed-conversion efficiency, or ecological traits that are better suited to CFS and mass-rearing?
• At what life stage should insects be harvested, particularly for metamorphosing species, based not only on their nutrient content, but also on their contributions to system productivity?

Within the CFS context, more specific questions emerge around population demography and resource management. Different insect species will likely differ in how their reproduction and growth respond to the volume, timing, and composition of available waste streams. This presents a challenge for maintaining viable populations capable of consistent waste processing and nutrient output across fluctuating conditions. For example, different feeds may influence individual growth (affecting the biomass available for harvest) and fecundity (impacting population persistence). These effects will likely vary by species, life stage, and rearing environment, and may introduce trade-offs between maximising output and sustaining insect populations over time. There are also open questions about how to optimise rearing conditions such as temperature, humidity, substrate type, and housing design, to enhance food conversion efficiency and insect health (Berggren, Jansson & Low, 2018). This includes identifying how rearing infrastructure should adapt to insects’ biological needs at different developmental stages, particularly for species that undergo complete metamorphosis (Cappellozza et al., 2019). Importantly, insect species differ significantly in their nutritional requirements, stress tolerances, and behavioural ecology. As with traditional livestock, these species should not be treated as interchangeable. Designing stable and productive insect-based systems will require species-specific knowledge of how to maintain health, support reproduction, and ensure functional roles under circular production conditions.

### Organism interactions within circular food systems

Although some studies suggest microbial communities are influenced by frass, the results show these relationships remain largely untested in CFS. Microbiota are essential drivers of biogeochemical cycling in ecosystems. For example, in crop production the soil microbiome is strongly shaped by local plant species and nutrient inputs (Poonam et al., 2023; Huang et al., 2024). While managing limiting elements in soils is a major goal for sustainable agriculture, there are still significant knowledge gaps regarding how soil nutrient dynamics operate in the context of CFS (Siddiqui et al., 2023). One critical priority is to examine how edible insect species contribute to nutrient recycling, including species-specific differences in their ability to extract nutrients from waste and partition them into body biomass *versus* excreted frass. These interactions are likely to be influenced by both the abiotic environment (*e.g.*, temperature, substrate quality) and biotic interactions (*e.g.*, with microbes), which in turn affect soil community structure and function. A better understanding of these relationships will help identify which plant–insect–microbe combinations are most compatible and productive within specific CFS designs. However, empirical data on species interactions within a CFS framework remain absent. This includes intra-trophic interactions (*e.g.*, potential competition or complementarity among insect species) and cross-trophic dynamics (*e.g.*, between insects, plants, and microbiota), especially when insect frass serves as both a nutrient and microbial substrate.

## Conclusions

### Ecological constraints of circular food systems

The preceding sections highlight several constraints that affect insect integration across system scales. Here, I synthesise these constraints to outline what makes ecological design for circular systems particularly complex. Building on the key knowledge gaps identified in the reviewed literature, the primary ecological and system-level challenges for integrating insects into circular food systems are outlined. Rather than restating the findings, I synthesise cross-cutting themes and propose future directions to guide research and system design. As highlighted in the results, few studies have examined full life-cycle performance of even the most common insect species used in food production (Berggren, Jansson & Low, 2018; Berggren, Jansson & Low, 2019). In a CFS, this knowledge gap is even more critical: not only must insects function as waste bioconverters, but their biology must be compatible with the specific constraints imposed by a CFS. One major constraint is the system-specific nature of biological waste. For instance, in crop production waste streams will depend on the crops being cultivated and may result in nutrient-homogenous, temporally irregular byproducts that may not align with the optimal feeding requirements of insect species. Waste utilisation, insect growth, and frass production are tightly interdependent in these systems (Fig. 1B). The timing, composition, and quality of waste directly influence insect physiology and the quality of the nutrients they recycle. For example, a CFS built on tomato and spinach cultivation might generate fibrous plant waste low in nitrogen and fatty acids. If house crickets (*A. domesticus*) are fed this material, they may experience reduced growth and survival due to insufficient dietary protein and fat. This could lead to lower frass output with poorer nutrient quality, which in turn could impair the productivity of the next plant cycle, creating a negative feedback loop across system components. This illustrates a broader challenge: identifying insect species that are not only capable of efficiently recycling waste into harvestable nutrient outputs, but that also exhibit resilient populations under variable feed quality and availability. Successful integration of insects into a CFS will depend on matching species’ nutritional needs and life-history traits with the timing, volume, and composition of system-generated waste.

In addition, rapid mass-rearing capabilities may be required to handle waste production surges, and husbandry systems must support key behaviours and life stages under system-specific conditions. Factors such as health, reproduction, and developmental plasticity must be assessed in context (Berggren, Jansson & Low, 2019; De Miranda et al., 2021b; De Miranda et al., 2021a). The review highlights substantial and urgent knowledge gaps that currently limit the implementation of insects as functional ecological agents in circular food systems. While many of these gaps fall within distinct disciplinary domains (*e.g.*, entomology, soil science, agronomy), effective CFS design will require cross-disciplinary collaboration to account for the ecological complexity and species interactions that underpin these systems. These findings underscore the need for targeted ecological research priorities (Fig. 3).

### Toward ecosystem-based circular food systems

Ecological interactions are crucial for predicting how nutrients will flow through the system and for ensuring system stability. Yet, due to a lack of foundational empirical studies, theoretical modelling of these interactions remains unreliable (Albalate-Ramírez et al., 2024). The transition from linear food systems to CFS poses major scientific and technical challenges. However, the potential to limit environmental degradation and achieve long-term sustainability makes this transformation essential. While insights from linear systems may provide an important foundation, a CFS must be understood as a simplified ecosystem composed of primary producers, consumers, decomposers, and recyclers that interact to support multi-trophic productivity. Successfully implementing such systems will require an ecosystem engineering approach, combining ecological understanding with transdisciplinary expertise. Insects are likely to become key actors in these systems due to their dual capacity to convert waste streams into consumable biomass and to produce nutrient-rich frass. Despite theoretical interest, as summarised in the results, current empirical knowledge about how to functionally integrate insects into a CFS remains limited. Expertise from entomology, soil science, agronomy, and systems ecology will all be required to design and evaluate such systems. Additionally, to ensure that circular systems are truly sustainable, research must also address their economic feasibility and social acceptability.

In summary, this review highlights the untapped ecological potential of insects in circular food systems and identifies key knowledge gaps that must be addressed to realise their full role as recyclers and nutrient providers. By synthesising both empirical and theoretical studies, this review offers a foundation for future research that treats food systems as engineered ecosystems where biological interactions, system flows, and sustainability targets are co-designed. Moving toward such systems will require transdisciplinary collaboration to integrate ecological principles into the redesign of food production. This proactive shift is essential not only to reduce environmental costs but to regenerate ecosystems through sustainable circularity.

## Supplemental Information

10.7717/peerj.21419/supp-1 Supplemental Information 1 PRISMA-style checklist

10.7717/peerj.21419/supp-2 Supplemental Information 2 Empirical studies identified in this review that assess the use of insects within circular food systems

10.7717/peerj.21419/supp-3 Supplemental Information 3 Reference list of all 63 studies included in the review of insects in circular food systems

## Additional Information and Declarations

### Data Availability

The following information was supplied regarding data availability: This is a Literature Review.
