# Supplementary material for: Integrating insects in circular food systems: evidence, gaps and research priorities
Source: PeerJ. 2026 Jul 10;14:e21419. doi: 10.7717/peerj.21419 (PMC13360746; doi:10.7717/peerj.21419)
Supplement: Supplemental Information 1 [file peerj-14-21419-s001.pdf]

Supplementary material

Table S1. PRISMA-style checklist

Section	Item	PRISMA Item Description	Location where item is reported
Title	1	Identify the manuscript as a review	Abstract
Abstract	2	Structured summary of review methods, scope, and findings	Abstract
Introduction	3	Rationale for the review	Introduction
Introduction	4	Explicit review question(s) or aim	End of Introduction
Methods	5	Study inclusion/exclusion criteria	Screening and inclusion criteria
Methods	6	Databases searched, search dates	Literature search
Methods	7	Full search strategy	Literature search
Methods	8	Screening process	Screening and inclusion criteria; Figure 2
Methods	9	What information was extracted	Eligibility assessment and study classification
Methods	10	Limitations of review process	Scope and Delimitation
Results	11	How results were synthesised	The state of knowledge
Results	12	Number and type of studies included	The state of knowledge
Discussion	13	Implications and research agenda	Final paragraph of Discussion
