# Supplementary material for: Integrating insects in circular food systems: evidence, gaps and research priorities
Source: PeerJ. 2026 Jul 10;14:e21419. doi: 10.7717/peerj.21419 (PMC13360746; doi:10.7717/peerj.21419)
Supplement: Supplemental Information 2 [file peerj-14-21419-s002.pdf]

## Supplementary material

**Table S2**: Empirical studies identified in this review that assess the use of insects within circular food systems. The table provides publication metadata and the insect species used in the study.

| Publication | Insect species |
|---|---|
| **Aboelkheir MG, Visconte LY, Oliveira GE, Toledo Filho RD, Souza Jr FG. 2019.** The biodegradative effect of *Tenebrio molitor* Linnaeus larvae on vulcanized SBR and tire crumb. *Science of the Total Environment* **649**:1075–1082. https://doi.org/10.1016/j.scitotenv.2018.08.228 | *Tenebrio molitor* |
| **Aziz M, Monticelli M, Radwan L, Abdelkader S, Bellini B, Buldrini G, Coluccia M, Carvino A, Elkady M, Signorini L, Orsini F. 2022.** Nurturing the soul at the urban garden: a project for Salus space, Bologna, Italy. *Acta Horticulturae* **1345**:473–480. https://doi.org/10.17660/ActaHortic.2022.1345.65 | *not stated* |
| **Beesigamukama D, Mochoge B, Korir N, Menale K, Muriithi B, Kidoido M, Kirscht H, Diiro G, Ghemoh CJ, Nakimbugwe D, Musyoka MW, Ekesi S, Tanga CM. 2022.** Economic and ecological values of frass fertiliser from black soldier fly agro-industrial waste processing. *Journal of Insects as Food and Feed* **8**:245–254. https://doi.org/10.3920/JIFF2021.0013 | *Hermetia illucens* |
| **Beesigamukama D, Subramanian S, Tanga CM. 2022.** Nutrient quality and maturity status of frass fertilizer from nine edible insects. *Scientific Reports* **12**:7182. https://doi.org/10.1038/s41598-022-11336-z | *Hermetia illucens, Tenebrio molitor, Scapsipedus icipe, Bombyx mori, Gryllus bimaculatus, Gonimbrasia krucki, Pachnoda sinuata, Schistocerca gregaria, Oryctes rhinoceros* |
| **Bruno D, Orlando M, Testa E, Miino MC, Pesaro G, Miceli M, Pollegioni L, Barbera V, Fasoli E, Draghi L, Baltrocchi APD, Ferronato N, Seri R, Maggi E, Caccia S, Casartelli M, Molla G, Galimberti MS, Toretta V, Vezzulii A, Tettamanti G. 2025.** Valorization of organic waste through black soldier fly: On the way of a real circular bioeconomy process. *Waste Management* **191**:123–134. https://doi.org/10.1016/j.wasman.2024.10.030 | *Hermetia illucens* |
| **Candian V, Tedeschi R. 2023.** Impact of the diet on the mortality and on gene expression of the antimicrobial peptide | *Tenebrio molitor* |

Tenecin 3 in *Tenebrio molitor* larvae infected by Beauveria bassiana. *Insects* **14**:359. https://doi.org/10.3390/insects14040359

**Chineme A, Assefa G, Herremans IM, Wylant B, Shumo M, Shoo A, Jabuligwe S, Yhedgo M. 2023.** Advancing circular economy principles through wild black soldier flies. AIMS Environmental Science 10:868–893. https://doi.org/10.3934/environsci.2023047 — *Hermetia illucens*

**Ebeneezar S, Tejpal CS, Jeena NS, Summaya R, Chandrasekar S, Sayooj P, Vijayagopal P. 2021.** Nutritional evaluation, bioconversion performance and phylogenetic assessment of black soldier fly (*Hermetia illucens*, Linn. 1758) larvae valorized from food waste. *Environmental Technology & Innovation* **23**:101783. https://doi.org/10.1016/j.eti.2021.101783 — *Hermetia illucens*

**Jones JJ, Shaw C, Chen TW, Staß CM, Ulrichs C, Riewe D, Kloas W, Geilfus CM. 2024.** Plant nutritional value of aquaculture water produced by feeding Nile tilapia (*Oreochromis niloticus*) alternative protein diets: A lettuce and basil case study. *Plants, People, Planet* **6**:362–380. https://doi.org/10.1002/ppp3.10457 — *Hermetia illucens*

**Jung H, Shin G, Park SB, Jegal J, Park SA, Park J, Oh DX, Kim HJ. 2023.** Circular waste management: Superworms as a sustainable solution for biodegradable plastic degradation and resource recovery. *Waste Management* **171**:568–579. https://doi.org/10.1016/j.wasman.2023.09.027 — *Zophobas atratus*

**Lalander C, Ermolaev E, Wiklicky V, Vinnerås B. 2020.** Process efficiency and ventilation requirement in black soldier fly larvae composting of substrates with high water content. *Science of the Total Environment* **729**:138968. https://doi.org/10.1016/j.scitotenv.2020.138968 — *Hermetia illucens*

**Monteiro dos Santos DK, Santana TM, de Matos Dantas F, Farias ABDS, Epifânio CMF, Prestes AG, da Fonesca CMF, Parisi G, Viegas EMM, Gonçalves LU. 2022.** Defatted black soldier fly larvae meal as a dietary ingredient for tambaqui (*Colossoma macropomum*): Digestibility, growth performance, haematological parameters, and carcass composition. *Aquaculture Research 53*:6762–6770. https://doi.org/10.1111/are.16143 — *Hermetia illucens*

**Paris N, Fortin A, Hotte N, Rasooli Zadeh A, Jain S, Hénault-Ethier L. 2024.** Developing an environmental assessment framework for an insect farm operating in a circular — *Tenebrio molitor*

economy: The case study of a Montréal (Canada) mealworm farm. *Journal of Cleaner Production* **460**:142450. https://doi.org/10.1016/j.jclepro.2024.142450

**Psarianos M, Fricke A, Altuntaş H, Baldermann S, Schreiner M, Schlüter OK. 2024.** Potential of house crickets *Acheta domesticus* L. (Orthoptera: Gryllidae) as a novel food source for integration in a co-cultivation system. *Future Foods* **9**:100332. https://doi.org/10.1016/j.fufo.2024.100332 — *Acheta domesticus*

**Randazzo B, Zarantoniello M, Gioacchini G, Cardinaletti G, Belloni A, Giorgini E, Faccenda F, Cerri R, Tibaldi E, Olivotto I. 2021.** Physiological response of rainbow trout (*Oncorhynchus mykiss*) to graded levels of *Hermetia illucens* or poultry by-product meals as single or combined substitute ingredients to dietary plant proteins. *Aquaculture* **538**:736550. https://doi.org/10.1016/j.aquaculture.2021.736550 — *Hermetia illucens*

**Ribeiro N, Costa R, Ameixa OM. 2022**. The influence of non-optimal rearing conditions and substrates on the performance of the black soldier fly (*Hermetia illucens*). *Insects* **13**:639. https://doi.org/10.3390/insects13070639 — *Hermetia illucens*

**Shaw C, Knopf K, Klatt L, Marin Arellano G, Kloas W. 2023.** Closing nutrient cycles through the use of system-internal resource streams: Implications for circular multitrophic food production systems and aquaponic feed development. *Sustainability* **15**:7374. https://doi.org/10.3390/su15097374 — *Hermetia illucens*

**Szopa D, Skrzypczak D, Izydorczyk G, Chojnacka K, Korczyński M, Witek-Krowiak A. 2023.** Evaluation of *Tenebrio molitor* protein hydrolysates as biostimulants improving plants growth and root architecture. *Journal of Cleaner Production* **401**:136812. https://doi.org/10.1016/j.jclepro.2023.136812 — *Tenebrio molitor*

**Van Dongen KCW, De Lange E, Van Asseldonk LLM, Zoet L, Van Der Fels-Klerx HJ. 2024.** Safety and transfer of veterinary drugs from substrate to black soldier fly larvae. *Animal* **18**:101214. https://doi.org/10.1016/j.animal.2024.101214 — *Hermetia illucens*

**Wang F, Zhao Q, Zhang L, Chen J, Wang T, Qiao L, Zhang L, Ding C, Yuan Y, Qi Z, Chen T. 2023.** Co-digestion of chicken manure and sewage sludge in black soldier fly larvae bioconversion system: bacterial biodiversity and nutrients quality of residues for biofertilizer application. *Environmental Science* — *Hermetia illucens*

*and Pollution Research* **30**:119804–119813. https://doi.org/10.1007/s11356-023-30717-z

**Walter A, Klammsteiner T, Gassner M, Heussler CD, Kapelari S, Schermer M, Insam H. 2020.** Black soldier fly school workshops as means to promote circular economy and environmental awareness. *Sustainability* **12**:9574. https://doi.org/10.3390/su12229574 — *Hermetia illucens*

**Zhang Z, Fan S, Jiang S, Deng Y, Xu B, Xiang F. 2024**. Effect of full-fat black soldier fly (*Hermetia illucens* L.) larvae on growth performance, immunological parameters, and gene expressions in zebrafish (*Danio rerio*). *International Aquatic Research* **16**:55–69. https://doi.org/10.22034/iar.2024.2003652.1570 — *Hermetia illucens*

**Zarantoniello M, de Oliveira AA, Sahin T, Freddi L, Torregiani M, Tucciarone I, Chemello G, Cardinaletti G, Gatto E, Parisi G, Bertolucci C, Riolo P, Nartea A, Gioacchini G, Olivotto I. 2023.** Enhancing rearing of European seabass (*Dicentrarchus labrax*) in aquaponic systems: Investigating the effects of enriched black soldier fly (*Hermetia illucens)* prepupae meal on fish welfare and quality traits. *Animals* 13:1921. https://doi.org/10.3390/ani13121921 — *Hermetia illucens*
