# Supplementary material for: Integrating insects in circular food systems: evidence, gaps and research priorities
Source: PeerJ. 2026 Jul 10;14:e21419. doi: 10.7717/peerj.21419 (PMC13360746; doi:10.7717/peerj.21419)
Supplement: Supplemental Information 3 [file peerj-14-21419-s003.pdf]

## Supplementary material

**Article S1**: Reference list of all 63 studies included in the review of insects in circular food systems. This include both empirical studies and theoretical or modelling-based contributions.

**Abro Z, Kassie M, Tanga C, Beesigamukama D, Diiro G. 2020.** Socio-economic and environmental implications of replacing conventional poultry feed with insect-based feed in Kenya. *Journal of Cleaner Production* **265**:121871. https://doi.org/10.1016/j.jclepro.2020.121871

**Aiking H, de Boer J. 2019.** Protein and sustainability–the potential of insects. *Journal of Insects as Food and Feed* **5**:3–7. https://doi.org/10.3920/JIFF2018.0011

**Aziz M, Monticelli M, Radwan L, Abdelkader S, Bellini B, Buldrini G, Coluccia M, Carvino A, Elkady M, Signorini L, Orsini F. 2022.** Nurturing the soul at the urban garden: a project for Salus space, Bologna, Italy. *Acta Horticulturae* **1345**:473–480. https://doi.org/10.17660/ActaHortic.2022.1345.65

**Beesigamukama D, Mochoge B, Korir N, Menale K, Muriithi B, Kidoido M, Kirscht H, Diiro G, Ghemoh CJ, Nakimbugwe D, Musyoka MW, Ekesi S, Tanga CM. 2022.** Economic and ecological values of frass fertiliser from black soldier fly agro-industrial waste processing. *Journal of Insects as Food and Feed* **8**:245–254. https://doi.org/10.3920/JIFF2021.0013

**Beesigamukama D, Subramanian S, Tanga CM. 2022.** Nutrient quality and maturity status of frass fertilizer from nine edible insects. *Scientific Reports* **12**:7182. https://doi.org/10.1038/s41598-022-11336-z

**Borrello M, Caracciolo F, Lombardi A, Pascucci S, Cembalo L. 2017.** Consumers' perspective on circular economy strategy for reducing food waste. *Sustainability* **9**:141. https://doi.org/10.3390/su9010141

**Bruno D, Orlando M, Testa E, Miino MC, Pesaro G, Miceli M, Pollegioni L, Barbera V, Fasoli E, Draghi L, Baltrocchi APD, Ferronato N, Seri R, Maggi E, Caccia S, Casartelli M, Molla G, Galimberti MS, Toretta V, Vezzulii A, Tettamanti G. 2025.** Valorization of organic waste through black soldier fly: On the way of a real circular bioeconomy process. *Waste Management* **191**:123–134. https://doi.org/10.1016/j.wasman.2024.10.030

**Candian V, Tedeschi R. 2023.** Impact of the diet on the mortality and on gene expression of the antimicrobial peptide Tenecin 3 in *Tenebrio molitor* larvae infected by Beauveria bassiana. *Insects* **14**:359. https://doi.org/10.3390/insects14040359

**Cattaneo A, Meneguz M, Dabbou S. 2023**. The fatty acid composition of black soldier fly larvae: the influence of feed substrate and applications in the feed industry. *Journal of Insects as Food and Feed* **10**:533–558. https://doi.org/10.1163/23524588-20230068

**Chineme A, Assefa G, Herremans IM, Wylant B, Shumo M, Shoo A, Jabuligwe S, Yhedgo M. 2023.** Advancing circular economy principles through wild black soldier flies. *AIMS Environmental Science* **10**:868–893. https://doi.org/10.3934/environsci.2023047

**Derler H, Lienhard A, Berner S, Grasser M, Posch A, Rehorska R. 2021.** Use them for what they are good at: Mealworms in circular food systems. *Insects* **12**:40. https://doi.org/10.3390/insects12010040

**DiGiacomo K, Leury BJ. 2019.** Insect meal: a future source of protein feed for pigs? *Animal* **13**:3022–3030. https://doi.org/10.1017/S1751731119001873

**Duncan AJ, Ayantunde A, Blummel M, Amole T, Padmakumar V, Moran D. 2023.** Applying circular economy principles to intensification of livestock production in Sub-Saharan Africa. *Outlook on Agriculture* **52**:327–338. https://doi.org/10.1177/00307270231199116

**Ebeneezar S, Tejpal CS, Jeena NS, Summaya R, Chandrasekar S, Sayooj P, Vijayagopal P. 2021.** Nutritional evaluation, bioconversion performance and phylogenetic assessment of black soldier fly (*Hermetia illucens*, Linn. 1758) larvae valorized from food waste. *Environmental Technology & Innovation* **23**:101783. https://doi.org/10.1016/j.eti.2021.101783

**Errico S, Verardi A, Sangiorgio P, Dimatteo S, Spagnoletta A, Moliterni S, Baldacchino F, Balducchi R. 2022.** *Tenebrio molitor*: innovative tool for food loss and waste valorization and biopolymers recovery. *Environmental Engineering and Management Journal* **21**:1641–1656. https://www.eemj.eu/index.php/EEMJ/article/view/4592

**Franzluebbers AJ, Martin G. 2022.** Farming with forages can reconnect crop and livestock operations to enhance circularity and foster ecosystem services. *Grass and Forage Science* **77**:270–281. https://doi.org/10.1111/gfs.12592

**Frasnetti E, Sadeqi H, Lamastra L. 2023.** Integrating insects into the agri-food system of northern Italy as a circular economy strategy. *Sustainable Production and Consumption* **43**:181–193. https://doi.org/10.1016/j.spc.2023.11.007

**Hasan Z, Lateef M. 2024.** Transforming food waste into animal feeds: an in-depth overview of conversion technologies and environmental benefits. *Environmental Science and Pollution Research* **31**:17951–17963. https://doi.org/10.1007/s11356-023-30152-0

**Jagtap S, Garcia-Garcia G, Duong L, Swainson M, Martindale W. 2021.** Codesign of food system and circular economy approaches for the development of livestock feeds from insect larvae. *Foods* **10**:1701. https://doi.org/10.3390/foods10081701

**Jones JJ, Shaw C, Chen TW, Staß CM, Ulrichs C, Riewe D, Kloas W, Geilfus CM. 2024.** Plant nutritional value of aquaculture water produced by feeding Nile tilapia (*Oreochromis niloticus*) alternative protein diets: A lettuce and basil case study. *Plants, People, Planet* **6**:362–380. https://doi.org/10.1002/ppp3.10457

**Jung H, Shin G, Park SB, Jegal J, Park SA, Park J, Oh DX, Kim HJ. 2023.** Circular waste management: Superworms as a sustainable solution for biodegradable plastic degradation and

resource recovery. *Waste Management* **171**:568–579. https://doi.org/10.1016/j.wasman.2023.09.027

**Kuan ZJ, Chan BKN, Gan SKE. 2022.** Worming the circular economy for biowaste and plastics: *Hermetia illucens*, *Tenebrio molitor*, and *Zophobas morio*. *Sustainability* **14**:1594. https://doi.org/10.3390/su14031594

**Lalander C, Vinnerås B. 2022.** Actions needed before insects can contribute to a real closed-loop circular economy in the EU. *Journal of Insects as Food and Feed* **8**:337–342. https://doi.org/10.3920/JIFF2022.x003

**Lalander C, Ermolaev E, Wiklicky V, Vinnerås B. 2020.** Process efficiency and ventilation requirement in black soldier fly larvae composting of substrates with high water content. *Science of the Total Environment* **729**:138968. https://doi.org/10.1016/j.scitotenv.2020.138968

**Lisboa HM, Nascimento A, Arruda A, Sarinho A, Lima J, Batista L, Dantas MF, Andrade R. 2024.** Unlocking the potential of insect-based proteins: Sustainable solutions for global food security and nutrition. *Foods* **13**:1846. https://doi.org/10.3390/foods13121846

**Monteiro dos Santos DK, Santana TM, de Matos Dantas F, Farias ABDS, Epifânio CMF, Prestes AG, da Fonesca CMF, Parisi G, Viegas EMM, Gonçalves LU. 2022.** Defatted black soldier fly larvae meal as a dietary ingredient for tambaqui (*Colossoma macropomum*): Digestibility, growth performance, haematological parameters, and carcass composition. *Aquaculture Research* **53**:6762–6770. https://doi.org/10.1111/are.16143

**Moruzzo R, Riccioli F, Espinosa Diaz S, Secci C, Poli G, Mancini S. 2021.** Mealworm (*Tenebrio molitor*): Potential and challenges to promote circular economy. *Animals* **11**:2568. https://doi.org/10.3390/ani11092568

**Ojha S, Bussler S, Schlüter OK. 2020.** Food waste valorisation and circular economy concepts in insect production and processing. *Waste Management* **118**:600–609. https://doi.org/10.1016/j.wasman.2020.09.010

**Paris N, Fortin A, Hotte N, Rasooli Zadeh A, Jain S, Hénault-Ethier L. 2024.** Developing an environmental assessment framework for an insect farm operating in a circular economy: The case study of a Montréal (Canada) mealworm farm. *Journal of Cleaner Production* **460**:142450. https://doi.org/10.1016/j.jclepro.2024.142450

**Pinotti L, Mazzoleni S, Moradei A, Lin P, Luciano A. 2023.** Effects of alternative feed ingredients on red meat quality: a review of algae, insects, agro-industrial by-products and former food products. *Italian Journal of Animal Science* **22**:695–710. https://doi.org/10.1080/1828051X.2023.2238784

**Piwowar A, Wolańska W, Orkusz A, Kapelko M, Harasym J. 2023.** Modelling the factors influencing Polish consumers' approach towards new food products on the market. *Sustainability* **15**:2818. https://doi.org/10.3390/su15032818

**Poveda J. 2021.** Insect frass in the development of sustainable agriculture. A review. *Agronomy for Sustainable Development* **41**:5. https://doi.org/10.1007/s13593-020-00656-x

**Psarianos M, Fricke A, Altuntaş H, Baldermann S, Schreiner M, Schlüter OK. 2024.** Potential of house crickets *Acheta domesticus* L. (Orthoptera: Gryllidae) as a novel food source for integration in a co-cultivation system. *Future Foods* **9**:100332. https://doi.org/10.1016/j.fufo.2024.100332

**Rahmann G, Grimm D. 2021.** Food from 458 m²—calculation for a sustainable, circular, and local land-based and landless food production system. *Organic Agriculture* **11**:187–198. https://doi.org/10.1007/s13165-020-00288-1

**Ranjbari M, Esfandabadi ZS, Quatraro F, Vatanparast H, Lam SS, Aghbashlo M, Tabatabaei M. 2022.** Biomass and organic waste potentials towards implementing circular bioeconomy platforms: A systematic bibliometric analysis. *Fuel* **318**:123585. https://doi.org/10.1016/j.fuel.2022.123585

**Randazzo B, Zarantoniello M, Gioacchini G, Cardinaletti G, Belloni A, Giorgini E, Faccenda F, Cerri R, Tibaldi E, Olivotto I. 2021.** Physiological response of rainbow trout (*Oncorhynchus mykiss*) to graded levels of *Hermetia illucens* or poultry by-product meals as single or combined substitute ingredients to dietary plant proteins. *Aquaculture* **538**:736550. https://doi.org/10.1016/j.aquaculture.2021.736550

**Ribeiro N, Costa R, Ameixa OM. 2022.** The influence of non-optimal rearing conditions and substrates on the performance of the black soldier fly (*Hermetia illucens*). *Insects* **13**:639. https://doi.org/10.3390/insects13070639

**Rumpold BA, Langen N. 2020.** Consumer acceptance of edible insects in an organic waste-based bioeconomy. *Current Opinion in Green and Sustainable Chemistry* **23**:14–18. https://doi.org/10.1016/j.cogsc.2020.03.007

**Shafer PJ, Chen YH, Reynolds T, von Wettberg EJB. 2022.** Farm to institution to farm: Circular food systems with native entomoculture. *Frontiers in Sustainable Food Systems* **5**:721985. https://doi.org/10.3389/fsufs.2021.721985

**Shah AA, Totakul P, Matra M, Cherdthong A, Hanboonsong Y, Wanapat M. 2022.** Nutritional composition of various insects and potential uses as alternative protein sources in animal diets. *Animal Bioscience* **35**:317–331. https://doi.org/10.5713/ab.21.0447

**Shaw C, Knopf K, Klatt L, Marin Arellano G, Kloas W. 2023.** Closing nutrient cycles through the use of system-internal resource streams: Implications for circular multitrophic food production systems and aquaponic feed development. *Sustainability* **15**:7374. https://doi.org/10.3390/su15097374

**Sokame BM, Runyu JC, Tonnang HE. 2024.** Integrating edible insect into circular agriculture for sustainable production. *Sustainable Production and Consumption* **52**:80–94. https://doi.org/10.1016/j.spc.2024.10.015

**Suárez MÁ, Gambuzzi E, Disla JMS, Castejón G, Poggiaroni G, Ling J. 2023.** ROOTS–Circular policies for changing the biowaste system. *Open Research Europe* **3**:78. https://doi.org/10.12688/openreseurope.15507.1

**Szopa D, Skrzypczak D, Izydorczyk G, Chojnacka K, Korczyński M, Witek-Krowiak A. 2023.** Evaluation of *Tenebrio molitor* protein hydrolysates as biostimulants improving plants growth and root architecture. *Journal of Cleaner Production* **401**:136812. https://doi.org/10.1016/j.jclepro.2023.136812

**Tanga CM, Egonyu JP, Beesigamukama D, Niassy S, Kimathi E, Magara HJO, Omuse ER, Subramanian S, Ekesi S. 2021.** Edible insect farming as an emerging and profitable enterprise in East Africa. *Current Opinion in Insect Science* **48**:64–71. https://doi.org/10.1016/j.cois.2021.09.007

**Tiboldo G, Arata L, Coderoni S. 2024.** Back to the future: Are consumers ready to eat insect-fed poultry food products from a circular farming system? An assessment for Italy. *Future Foods* **9**:100290. https://doi.org/10.1016/j.fufo.2023.100290

**Torok VA, Luyckx K, Lapidge S. 2021.** Human food waste to animal feed: opportunities and challenges. *Animal Production Science* **62**:1129–1139. https://doi.org/10.1071/AN20631

**Turmel MS, Speratti A, Baudron F, Verhulst N, Govaerts B. 2015.** Crop residue management and soil health: A systems analysis. *Agricultural Systems* **134**:6–16. https://doi.org/10.1016/j.agsy.2014.05.009

**Valoppi F, Agustin M, Abik F, Morais de Carvalho D, Sithole J, Bhattarai M, Varis JJ, Arzami ANAB, Pulklinen E, Mikkonen KS. 2021.** Insight on current advances in food science and technology for feeding the world population. *Frontiers in Sustainable Food Systems* **5**:626227. https://doi.org/10.3389/fsufs.2021.626227

**Van Dongen KCW, De Lange E, Van Asseldonk LLM, Zoet L, Van Der Fels-Klerx HJ. 2024.** Safety and transfer of veterinary drugs from substrate to black soldier fly larvae. *Animal* **18**:101214. https://doi.org/10.1016/j.animal.2024.101214

**Van Raamsdonk LWD, Van der Fels-Klerx HJ, De Jong J. 2017.** New feed ingredients: the insect opportunity. *Food Additives & Contaminants: Part A* **34**:1384–1397. https://doi.org/10.1080/19440049.2017.1306883

**Voulgari-Kokota A, van Loon MS, Bovenkerk B. 2023.** Insects as mini-livestock: Considering insect welfare in feed production. *NJAS: Impact in Agricultural and Life Sciences* **95**:2191797. https://doi.org/10.1080/27685241.2023.2191797

**Walter A, Klammsteiner T, Gassner M, Heussler CD, Kapelari S, Schermer M, Insam H. 2020.** Black soldier fly school workshops as means to promote circular economy and environmental awareness. *Sustainability* **12**:9574. https://doi.org/10.3390/su12229574

**Wang F, Zhao Q, Zhang L, Chen J, Wang T, Qiao L, Zhang L, Ding C, Yuan Y, Qi Z, Chen T. 2023**. Co-digestion of chicken manure and sewage sludge in black soldier fly larvae

bioconversion system: bacterial biodiversity and nutrients quality of residues for biofertilizer application. *Environmental Science and Pollution Research* **30**:119804–119813. https://doi.org/10.1007/s11356-023-30717-z

**Weidner T, Yang A. 2020.** The potential of urban agriculture in combination with organic waste valorization: Assessment of resource flows and emissions for two European cities. *Journal of Cleaner Production* **244**:118490. https://doi.org/10.1016/j.jclepro.2019.118490

**Zarantoniello M, de Oliveira AA, Sahin T, Freddi L, Torregiani M, Tucciarone I, Chemello G, Cardinaletti G, Gatto E, Parisi G, Bertolucci C, Riolo P, Nartea A, Gioacchini G, Olivotto I. 2023**. Enhancing rearing of European seabass (*Dicentrarchus labrax*) in aquaponic systems: Investigating the effects of enriched black soldier fly (*Hermetia illucens*) prepupae meal on fish welfare and quality traits. *Animals* **13**:1921. https://doi.org/10.3390/ani13121921

**Zhang Z, Fan S, Jiang S, Deng Y, Xu B, Xiang F. 2024.** Effect of full-fat black soldier fly (*Hermetia illucens* L.) larvae on growth performance, immunological parameters, and gene expressions in zebrafish (*Danio rerio*). *International Aquatic Research* **16**:55–69. https://doi.org/10.22034/iar.2024.2003652.1570

**Zhang ZQ, Chen SC, Xiao JH, Huang DW. 2024.** State-of-the-art review of edible insect: From bioactives, pretreatment to enrichment. *Food Bioscience* **59**:103879. https://doi.org/10.1016/j.fbio.2024.103879
